# Dexamethasone added to local anesthetics in ultrasound-guided transversus abdominis plain (TAP) block for analgesia after abdominal surgery: A systematic review and meta-analysis of randomized controlled trials

**DOI:** 10.1371/journal.pone.0209646

**Published:** 2019-01-08

**Authors:** Donghang Zhang, Cheng Zhou, Dang Wei, Long Ge, Qian Li

**Affiliations:** 1 Department of Anesthesiology, West China Hospital, Sichuan University, Chengdu, Sichuan, China; 2 Lab of Anesthesia & Critical Care Medicine, Translational Neuroscience Center, West China Hospital, Sichuan University, Chengdu, Sichuan, China; 3 Evidence-Based Medicine Center, School of Basic Medical Sciences, Lanzhou University, Lanzhou, Gansu, China; Cleveland Clinic Lerner College of Medicine of Case Western Reserve University, UNITED STATES

## Abstract

**Objective:**

To evaluate the analgesic efficacy of dexamethasone added to local anesthetics in ultrasound-guided transversus abdominis plane (TAP) block for the patients after abdominal surgery.

**Methods:**

PubMed, CENTRAL, EMBASE, Web of science were searched to identify eligible randomized controlled trials (RCTs) that compared dexamethasone added to local anesthetics in ultrasound-guided TAP block with control for postoperative analgesia in adult patients undergoing abdominal surgery. Primary outcomes included postoperative pain intensity, the time to the first request for additional analgesics, and opioid consumption over 24 h after surgery. Secondary outcome was the incidence of postoperative nausea and vomiting. Analysis was performed by RevMan 5.3 software and the quality of evidence was rated using GRADE (Grading of Recommendations, Assessment, Development and Evaluation) approach.

**Results:**

Nine RCTs involving 575 patients were included. Compared to the control, dexamethasone added to local anesthetics in ultrasound-guided TAP block significantly decreased visual analogue scale (VAS) scores at rest at 4h (mean difference [MD] = -1.01; 95% confidence intervals [CI], -1.29 to -0.73; P<0.00001; moderate quality of evidence), 6h (MD = -1.21; 95% CI, -1.74 to -0.69; P<0.00001; low quality of evidence), and 12h after surgery (MD = -0.79; 95% CI, -0.97 to -0.60; P<0.00001; moderate quality of evidence). No difference was found at 2h (MD = -0.64; 95% CI, -1.35 to 0.08; P = 0.08; low quality of evidence) and 24 h (MD = -0.41; 95% CI, -0.91 to 0.09; P = 0.11; moderate quality of evidence) in VAS scores. The time to the first request for additional analgesics was prolonged in the dexamethasone group (MD = 3.08; 95% CI, 2.37 to 3.78; P<0.00001; moderate quality of evidence). Opioid consumption over 24 h after surgery was also reduced (MD = -5.42; 95% CI, -8.20 to -2.63; P = 0.0001; low quality of evidence). Meanwhile, the incidence of postoperative nausea and vomiting was significantly decreased in the dexamethasone group (risk ratios [RR] = 0.40; 95% CI, 0.28 to 0.58; P<0.00001; high quality of evidence). No complications were reported in all the included studies.

**Conclusions:**

Dexamethasone added to local anesthetics in ultrasound-guided TAP block was a safe and effective strategy for postoperative analgesia in adult patients undergoing abdominal surgery.

## Introduction

Transversus abdominis plane (TAP) block is widely used in abdominal surgery for postoperative analgesia[1]. Compared with placebo or no TAP block, TAP block can reduce pain scores, opioid consumption, and the incidence of opioid-related complications after abdominal surgery[2]. TAP block is therefore suggested as part of the multimodal analgesia to enhance recovery after abdominal surgery[3–5]. Ultrasound-guided TAP block, first described in 2007 by Hebbard et al., has significantly improved the performance and success rate of this technique[6]. Generally, single-shot injection of local anesthetics can provide the analgesic duration for 4~12 hours after surgery[7]. Early studies indicate that the addition of dexamethasone to local anesthetics can prolong the analgesic duration in peripheral nerve blocks[8, 9]. The analgesic efficacy of dexamethasone added to local anaesthetics in TAP block after surgery has been explored recently, but the results are inconsistent[7, 10]. It’s thus worthwhile to perform a meta-analysis of RCTs to determine the efficacy of dexamethasone used for TAP block during the postoperative period of abdominal surgery in adult patients.

## Materials and methods

### Search strategy

Two authors (D.Z. and D.W.) independently searched PubMed, Cochrane Central Register of Controlled Trials (CENTRAL), EMBASE, and Web of science from the first record to August 20, 2018 using the following terms: “transversus abdominis plane block or TAP block” and “dexamethasone”. English-language restriction was applied. The search strategy conducted for PubMed is presented in the S1 Appendix. Additional studies were retrieved by reviewing the references of the relevant articles.

### Inclusion and exclusion

Inclusion criteria: (a) Design: randomized controlled trials (RCTs); (b) Population: adult patients undergoing abdominal surgery; (c) Intervention: ultrasound-guided TAP block using local anesthetics + dexamethasone (dexamethasone group); (d) Control: ultrasound-guided TAP block using local anesthetics + saline or nothing (control group); and (e) Primary outcomes: pain scores evaluated with visual analogue scale (VAS) or numeric rating scale (NRS), the time to the first request for additional analgesics (TFA), and opioid consumption over 24 h after surgery; Secondary outcome: the incidence of postoperative nausea and vomiting (PONV). Reviews, conference abstracts, letters, retrospective or case series, and studies of pediatric surgery were all excluded.

### Study selection

Two authors (D.Z. and D.W.) independently reviewed the identified studies. Full-text of potentially relevant articles were retrieved after screening titles and abstracts for eligibility. Disagreements were resolved by discussion with another author (L.G.).

### Data extraction

Two authors (D.Z. and D.W.) independently extracted the following data from eligible studies: authors, publication year, sample number, administration, types of surgery and anesthesia, and outcomes. Additional data were sought from the corresponding authors through email. Pain scores evaluated by the 0–100 mm visual analogue scale (VAS) were converted to the 0~10cm (0: no pain, 10: worst imaginable pain) scale. Numeric rating scale was regarded equivalent to visual analogue scale. Opioid consumption was all transformed to morphine-equivalent consumption (morphine 10 mg = tramadol 100 mg, i.v.)[11]. When data was presented using the median and range, an attempt was made to contact the author for the original data. If there was no respond, the median and range were converted to the mean and standard deviation[12]. Two interventions groups in one study were combined into one single intervention group[13]. We tried to contact the authors if pain scores were not reported at rest or on movement, and pain scores were assumed to be at rest if there was no reply. Disagreements were resolved by discussion with another author (L.G.).

### Risk of bias assessment

Two authors (D.Z. and D.W.) independently assessed the quality of included studies using the Cochrane Collaboration’s tool[13], which consists of six items as the following: (a) random sequence generation (selection bias); (b) allocation concealment (selection bias); (c) blinding of participants and personnel (performance bias); (d) blinding of outcome assessment (detection bias); (e) incomplete outcome data (attrition bias); and (f) selective reporting(reporting bias). The estimated risk of bias for each item was rated as ‘low’, ‘unclear’, or ‘high’. Disagreements were resolved by discussion with another author (L.G.).

### Statistical analysis

Data analysis was performed by RevMan 5.3 software (The Nordic Cochrane Centre, The Cochrane Collaboration, 2014.). Data were combined if an outcome was reported at least in two studies. Continuous data were summarized as weighted mean differences (MD) with 95% confidence intervals (CI). Dichotomous data were summarized as risk ratios (RR) and 95% CI. Considering the clinical heterogeneity (e.g. type of surgery, use of local anesthetics, amount of dexamethasone, kind of anesthesia), a random-effect model was used for all analysis. Statistical heterogeneity was assessed using the I^2^ test. Significant heterogeneity was considered to be present when I^2^ statistic was > 50%. P < 0.05 was considered to be statistically significant. The quality of evidence was judged using the GRADE approach[14] and rated as ‘high’, ‘moderate’, ‘low’, or ‘very low’. The results of GRADE were presented in a “Summary of findings” table.

## Results

### Characteristics of included studies

One hundred and seven studies were identified initially, to which 3 studies were obtained from the reference list of relevant studies, 40 duplicates were removed. From the 67 records left, 53 were excluded by screening titles and abstracts (13 reviews, conference abstracts or letters, 8 retrospective or case series, 5 duplicates, 27 not comparing TAP block with TAP block combined dexamethasone), leaving 14 potentially relevant studies for full text review. Five studies were subsequently excluded according to the inclusion criteria (1 case report,3 conference abstracts and 1 not English-language). Finally, 9 RCTs [7, 10, 15–21] involving 575 patients were included into this meta-analysis. Three hundred and five patients received TAP block using local anesthetics + dexamethasone and other 270 patients were served as controls.

**Fig 1:**
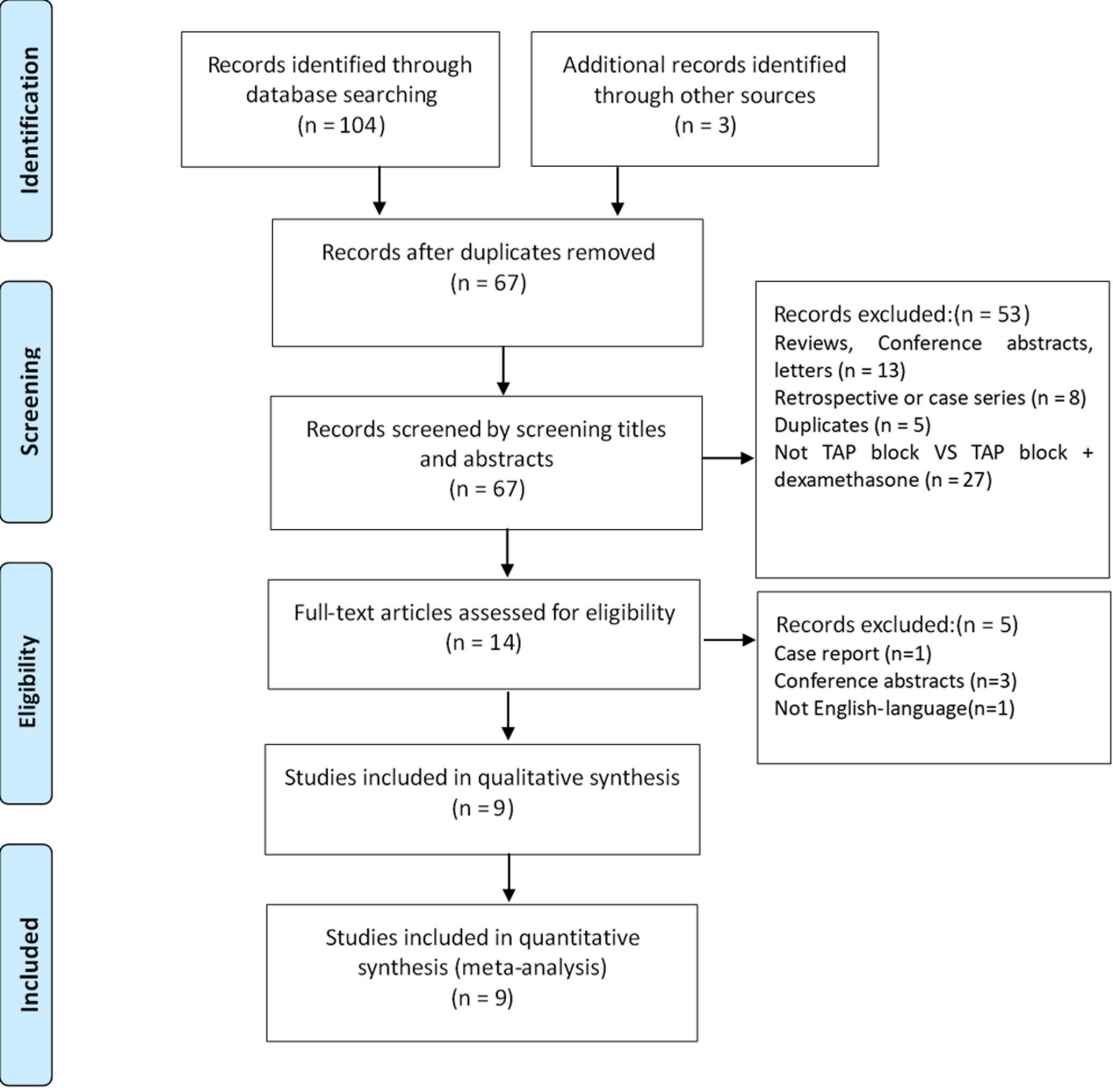
The flow diagram of study selection.

The flow diagram for the selection was shown in Fig 1. The characteristics of the included studies were shown in Table 1, and the risk of bias of the included studies was shown in Table 2.

**Table 1:** Characteristics of included trials.

Study	Group	Treatment	Surgery	Anesthesia	Postoperative analgesia	Outcomes
Akkaya 2014	Control (n = 21) Dexamethasone (n = 21)	After the surgery Bilateral 30 ml 0.25% levobupivacaine and 2 ml 0.9% NaCl or 2 ml dexamethasone (8 mg)	Caesarean section	Spinal anaesthesia	Tramadol 50 mg IV if needed	TFA, pain scores, total analgesic consumption, PONV
Ammar 2012	Control (n = 30) Dexamethasone (n = 30)	After the induction Bilateral 20 mL of bupivacaine hydrochloride 0.25% and 2 mL saline 0.9% or 2 mL dexamethasone (8 mg)	Abdominal hysterectomy	General anesthesia	Acetaminophen 1 g IV every 6 h during first 24 h after surgery, PCA morphine bolus 1 mg IV if needed.	TFA, pain scores, morphine consumption, PONV
Deshpande 2017	Control (n = 30) Dexamethasone (n = 30)	At the end of surgery Bilateral TAP block using 20 ml of 0.5% ropivacaine and 1 ml of 0.9% saline or 4 mg dexamethasone	Abdominal Hysterectomy	Spinal anesthesia	Tramadol 1 mg/kg IV on patient's demand or when VAS >4.	TFA, total analgesic consumption, PONV
Huang 2016	Control (n = 20) Dexamethasone (n = 20)	After the induction Bilateral TAP block with 15 mL of 0.375% ropivacaine or 1 mL of dexamethasone (5mg)	Laparoscopic cholecystectomy	General anesthesia	Parecoxib 40 mg IV before entering PACU, 20–40 mg at 6–12 hours intervals if needed, sufentanil 5–10 μg IV when VAS score≥4	TFA, pain scores, total analgesic consumption, and adverse effects.
Kartalov 2015	Control (n = 30) Dexamethasone (n = 30)	After the induction Unilateral TAP block with 25 ml of 0.5% ropivacaine or 4 mg dexamethasone	Open inguinal hernia repair	General anesthesia	Paracetamol 1g IV every 6 hours, morphine 0.05 mg/kg IV if VAS > 3 and if paracetamol had been administered less than 6 hours before.	Pain scores, the total morphine consumption
Wegner 2017	Control (n = 41) Dexamethasone (n = 41)	Immediately following surgery Unilateral 20 mL ropivacaine 0.2% combined with saline or 8 mg dexamethasone	Inguinal hernia repair and spermatocelectomy	General anesthesia	No details provided	Pain scores, PONV
El Sharnouby 2015	Control (n = 33) Dexamethasone4 (n = 34) Dexamethasone8 (n = 34)	At the end of surgery Bilateral 20 mL of bupivacaine hydrochloride 0.25% + 2 mL saline 0.9% or 1 mL saline 0.9% and 1 mL dexamethasone (4 mg) or 2 mL dexamethasone (8 mg)	laparoscopic vertical banded gastroplasty	General anesthesia	Paracetamol 1g IV every 6h during first 24 h after surgery, meperidine hydrochloride (50 mg) IV if needed.	TFA, pain scores, total analgesic consumption, PONV
Sachdeva 2016	Control (n = 35) Dexamethasone (n = 35)	At the end of surgery Bilateral 20 mL ropivacaine 0.2% combined with saline or 4 mg dexamethasone	Cesarean section	Spinal anesthesia	Tramadol 100 mg IV If VAS >3 even after 30min of receiving diclofenac1.5 mg/kg.	TFA, analgesic consumption, PONV
Sharma 2018	Control (n = 30) Dexamethasone (n = 30)	After the surgery Unilateral 20 mL ropivacaine 0.5% combined with saline or 8 mg dexamethasone	Inguinal hernia repair	Spinal anesthesia	Tramadol 2mg/kg IV if VAS>4 or on patient's demand	TFA, pain scores, total analgesic consumption, PONV

TAP = transversus abdominis plane; VAS = visual analog scale; PCA = patient controlled analgesia; IV = intravenous; TFA = the time to the first request for additional analgesics; PONV = postoperative nausea and vomiting.

**Table 2:** Risk of bias of included trials.

Study	Random sequence generation	Allocation concealment	Blinding of participants and personnel	Blinding of outcome assessment	Incomplete outcome data	Selective reporting
Akkaya,2014	Low	Low	Low	Low	Low	Low
Ammar,2012	Low	Low	Low	Low	Low	Low
Deshpande,2017	Low	Low	Low	Low	Low	Low
Huang,2016	Unclear	Unclear	Low	Low	Low	Low
Kartalov,2015	Unclear	Low	Low	Low	Low	Low
Wegner,2017	Low	Unclear	Low	Unclear	Low	Low
El Sharnouby,2015	Low	Low	Low	Low	Low	Low
Sachdeva,2016	Low	Low	Low	Unclear	Low	Low
Sharma,2018	Low	Low	Low	Low	Low	Low

Low = low risk of bias; Unclear = unclear risk of bias

The included studies were published from 2012 to 2018, performed in Turkey, Egypt, India, China, Macedonia, and America, respectively. Five studies[7, 16–19] were performed under general anesthesia while four studies[10, 15, 20, 21] were performed under spinal anesthesia. The type of surgery included transabdominal hysterectomy (n = 2), caesarean section (n = 2), laparoscopic cholecystectomy (n = 1), open inguinal hernia repair (n = 2), inguinal hernia repair and spermatocelectomy (n = 1), and laparoscopic vertical banded gastroplasty (n = 1). The dose of dexamethasone covered 4mg (n = 4), 8mg (n = 5) and 5mg (n = 1). The local anesthetic for TAP block was ropivacaine in six studies[7, 15–17, 20, 21], bupivacaine in two studies[18, 19], and levobupivacaine in one study[10]. Six studies[7, 10, 15, 19–21] performed the TAP block after surgery, and others[16–18] performed the TAP block after anesthesia induction.

### Results of meta-analyses

#### VAS scores at rest at 2 hours after surgery

Four studies[10, 15, 17, 21] reported VAS scores at rest at 2 h after surgery. No significant difference was found in VAS pain scores at rest at 2 h in the dexamethasone group compared with the control group (MD = -0.64; 95% CI, -1.35 to 0.08; I^2^ = 97%; P = 0.08) (Table 3).

**Table 3:** Pain scores (VAS) at rest at 5 different time points after surgery for the comparison of dexamethasone and control.

Time points	Studies, n	Patients, n	MD (95% CI)	p value	I^2^ test, %
2 h	4	222	-0.64(-1.35, 0.08)	0.08	97
4 h	4	222	-1.01(-1.29, -0.73)	<0.00001	16
6 h	4	220	-1.21(-1.74, -0.69)	<0.00001	71
12 h	6	344	-0.79(-0.97, -0.60)	<0.00001	0
24 h	6	344	-0.41(-0.91, 0.09)	0.11	84

MD = mean difference; CI = confidence interval; VAS = visual analogue scale.

#### VAS scores at rest at 4 hours after surgery

Four studies[10, 15, 17, 21] reported VAS scores at rest at 4 h after surgery. Pooled results showed a significant reduction in VAS pain scores at rest at 4 h in the dexamethasone group compared with the control group (MD = -1.01; 95% CI, -1.29 to -0.73; I^2^ = 16%; P<0.00001) (Table 3).

#### VAS scores at rest at 6 hours after surgery

Four studies[15–17, 21] reported VAS scores at rest at 6 h after surgery. Pooled results showed a significant reduction in VAS pain scores at rest at 6 h in the dexamethasone group compared with the control group (MD = -1.21; 95% CI, -1.74 to -0.69; I^2^ = 71%; P<0.00001) (Table 3).

#### VAS scores at rest at 12 hours after surgery

Six studies[7, 10, 15–17, 21] reported VAS scores at rest at 12 h after surgery. Pooled results showed a significant reduction in VAS pain scores at rest at 12 h in the dexamethasone group compared with the control group (MD = -0.79; 95% CI, -0.97 to -0.60; I^2^ = 0%; P<0.00001) (Table 3).

#### VAS scores at rest at 24 hours after surgery

Six studies[7, 10, 15–17, 21] reported VAS scores at rest at 24 h after surgery. Pooled results showed no difference in VAS pain scores at rest at 24 h in the dexamethasone group compared with the control group (MD = -0.41; 95% CI, -0.91 to 0.09; I^2^ = 84%; P = 0.11) (Table 3).

Subgroup analysis was conducted according to the timing of administration. No significant difference was shown between two groups when TAP block performed preoperatively (MD = -0.68; 95% CI, -1.45 to 0.09; I^2^ = 75%; P = 0.08) or postoperatively (MD = -0.16; 95% CI, -0.46 to 0.15; I^2^ = 18%; P = 0.33).

#### VAS scores on movement after surgery

One study[18] reported VAS scores on movement after surgery. The pain VAS score was significantly lower at 2 h (MD = -2.32; 95% CI, -2.72 to -1.92; P<0.00001), 4 h (MD = -1.89; 95% CI, -2.24 to -1.54; P<0.00001) and 12 h (MD = -0.97; 95% CI, -1.28 to -0.66; P<0.00001) in the dexamethasone group. No significant difference was found in VAS pain scores at 24 h between the two groups (MD = -0.18; 95% CI, -0.48 to 0.12; P = 0.24).

#### The time to the first request for additional analgesics (TFA)

**Fig 2:**
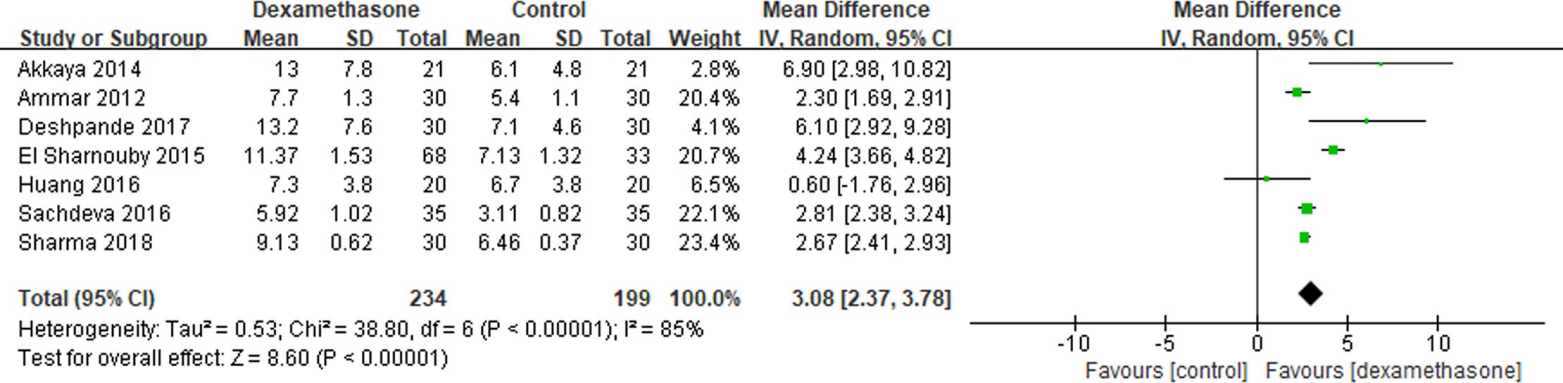
Meta-analysis of TFA. TFA = the time to the first request for additional analgesics.

Seven studies[10, 15, 16, 18–21] reported the TFA. Pooled results showed that TFA was prolonged significantly in the dexamethasone group compared with the control group (MD = 3.08; 95% CI, 2.37 to 3.78; I^2^ = 85%; P<0.00001) (Fig 2).

Subgroup analysis was conducted according to the timing of administration. A significant prolongation was shown in the dexamethasone group when TAP performed preoperatively (MD = 1.85; 95% CI, 0.37 to 3.32; I^2^ = 47%; P = 0.01) or postoperatively (MD = 3.54; 95% CI, 2.68 to 4.39; I^2^ = 87%; P<0.00001).

#### Morphine consumption over 24 hours after surgery

**Fig 3:**
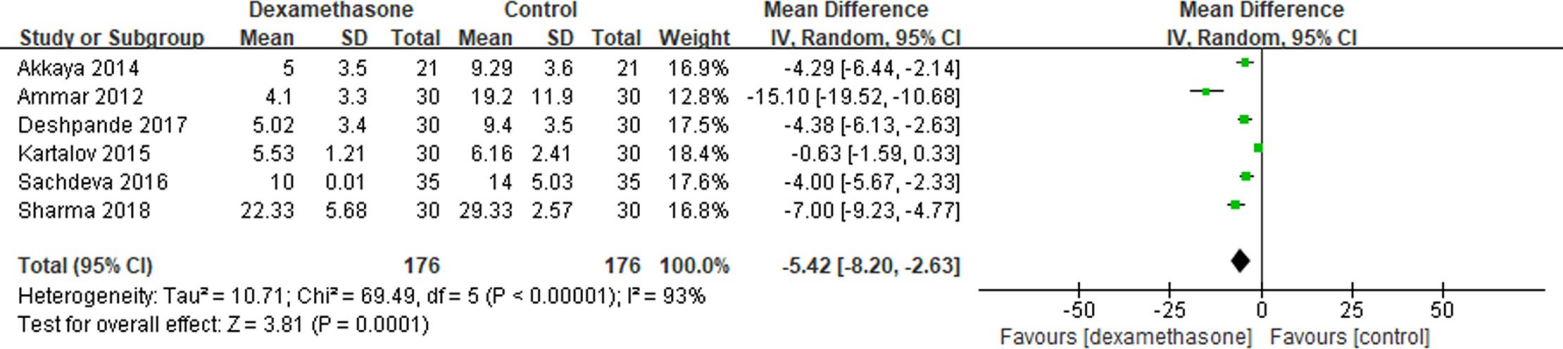
Meta-analysis of morphine consumption over 24 h after surgery.

Six studies[10, 15, 17, 18, 20, 21] reported morphine consumption over 24 h after surgery. Pooled results showed a significant reduction in morphine consumption over 24 hours in the dexamethasone group compared with the control group (MD = -5.42; 95% CI, -8.20 to -2.63; I^2^ = 93%; P = 0.0001) (Fig 3).

Subgroup analysis was conducted according to the timing of administration. No significant difference was shown between two groups when TAP block performed preoperatively (MD = -7.70; 95% CI, -21.87 to 6.48; I^2^ = 97%; P = 0.29). A significant reduction was shown in the dexamethasone group when TAP performed postoperatively (MD = -4.79; 95% CI, -6.04 to -3.55; I^2^ = 40%; P<0.00001).

#### The incidence of PONV over 24 hours after surgery

**Fig 4:**
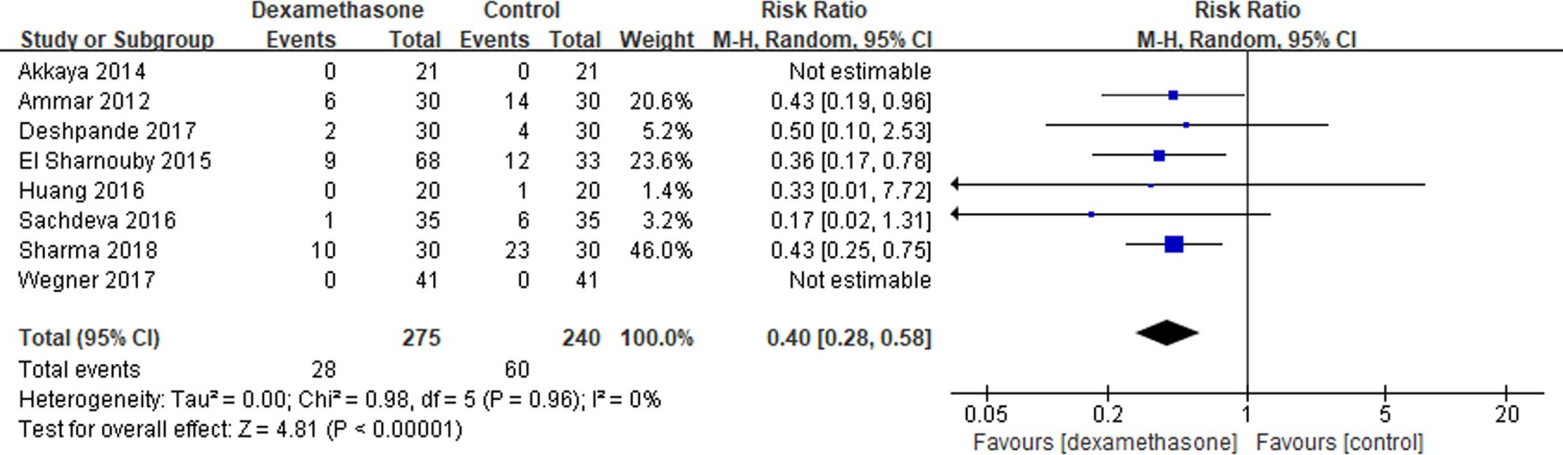
Meta-analysis of the incidence of PONV over 24 h after surgery. PONV = postoperative nausea and vomiting.

Eight studies[7, 10, 15, 16, 18–21] reported the incidence of PONV at 24 h after surgery. Pooled results showed a lower incidence of PONV in the dexamethasone group compared with the control group (RR = 0.40; 95% CI, 0.28 to 0.58; I^2^ = 0%; P<0.00001) (Fig 4).

Subgroup analysis was conducted according to the timing of administration. A significant reduction was shown in the dexamethasone group when TAP block performed preoperatively (RR = 0.42; 95% CI, 0.19 to 0.93; I^2^ = 0%; P = 0.03) or postoperatively (RR = 0.40; 95% CI, 0.26 to 0.61; I^2^ = 0%; P<0.0001).

### Publication bias

Funnel plots for publication bias could not be reliably tested because of the small number of included studies.

### Quality of evidence

The quality of evidence for all outcomes was shown in Table 4.

**Table 4:** Summary of findings.

Outcomes	Number of patients (studies)	Quality of evidence	Relative effect (95% CI)	Anticipated absolute effects	
				Risk with placebo	Risk difference with outcomes
VAS 2h at rest	222 (4 RCTs)	⨁⨁◯◯ LOW ^a^^,^^b^^,^^c^	-	The mean VAS 2h at rest was **0**	MD **0.64 lower** (1.35 lower to 0.08 higher)
VAS 4h at rest	222 (4 RCTs)	⨁⨁⨁◯ MODERATE ^b^	-	The mean VAS 4h at rest was **0**	MD **1.01 lower** (1.29 lower to 0.73 lower)
VAS 6h at rest	220 (4 RCTs)	⨁⨁◯◯ LOW ^a^^,^^b^	-	The mean VAS 6h at rest was **0**	MD **1.21 lower** (1.74 lower to 0.69 lower)
VAS 12h at rest	344 (6 RCTs)	⨁⨁⨁◯ MODERATE ^b^	-	The mean VAS 12h at rest was **0**	MD **0.79 lower** (0.97 lower to 0.6 lower)
VAS 24h at rest	344 (6 RCTs)	⨁⨁⨁◯ MODERATE ^b^^,^^c^	-	The mean VAS 24h at rest was **0**	MD **0.41 lower** (0.91 lower to 0.09 higher)
VAS 2h on movement	60 (1 RCT)	⨁⨁⨁◯ MODERATE ^b^	-	The mean VAS 2h on movement was **0**	MD **2.32 lower** (2.72 lower to 1.92 lower)
VAS 4h on movement	60 (1 RCT)	⨁⨁⨁◯ MODERATE ^b^	-	The mean VAS 4h on movement was **0**	MD **1.89 lower** (2.24 lower to 1.54 lower)
VAS 12h on movement	60 (1 RCT)	⨁⨁⨁◯ MODERATE ^b^	-	The mean VAS 12h on movement was **0**	MD **0.97 lower** (1.28 lower to 0.66 lower)
VAS 24h on movement	60 (1 RCT)	⨁⨁⨁◯ MODERATE ^b^^,^^c^	-	The mean VAS 24h on movement was **0**	MD **0.18 lower** (0.48 lower to 0.12 higher)
TFA	433 (7 RCTs)	⨁⨁⨁◯ MODERATE ^a^	-	The mean TFA was **0**	MD **3.08 higher** (2.37 higher to 3.78 higher)
morphine consumption	352 (6 RCTs)	⨁⨁◯◯ LOW ^a^^,^^b^	-	The mean morphine consumption was **0**	MD **5.42 lower** (8.2 lower to 2.63 lower)
nausea and vomiting	515 (8 RCTs)	⨁⨁⨁⨁ HIGH	**RR 0.40** (0.28 to 0.58)	250 per 1,000	**150 fewer per 1,000** (180 fewer to 105 fewer)

a. There is evidently statistical heterogeneity among the included studies.

b. The sample size is less than optimal information sample size.

c. The 95% confident interval of pooled effect estimate is large which includes the point of equal effect.

The risk in the intervention group (and its 95% confidence interval) is based on the assumed risk in the comparison group and the relative effect of the intervention (and its 95% CI).

**CI:** Confidence interval; **RR:** Risk ratio; **MD:** Mean difference

GRADE Working Group grades of evidence

**High quality:** We are very confident that the true effect lies close to that of the estimate of the effect

**Moderate quality:** We are moderately confident in the effect estimate: The true effect is likely to be close to the estimate of the effect, but there is a possibility that it is substantially different

**Low quality:** Our confidence in the effect estimate is limited: The true effect may be substantially different from the estimate of the effect

**Very low quality:** We have very little confidence in the effect estimate: The true effect is likely to be substantially different from the estimate of effect

## Discussion

This meta-analysis indicates that the addition of dexamethasone to local anesthetics for ultrasound-guided TAP block can result in decreased intensity of postoperative pain at rest at 4,6,12h for the patients after abdominal surgeries (low or moderate quality evidence). The time to the first request for additional analgesics is prolonged in dexamethasone group (moderate quality evidence). Morphine consumption over 24 h after surgery is significantly reduced (low quality evidence). There is a significant reduction in PONV when dexamethasone used (high quality evidence).

Enhanced Recovery After Surgery (ERAS), firstly proposed by Kehlet[22], is now applied to most surgery fields. ERAS is a multimodal perioperative care pathway designed to decrease morbidity, length of hospital stay, and promote postoperative recovery[23]. However, postoperative pain is one of the most undesirable consequences for the patients[24]. The pain after abdominal surgery is largely related to somatic pain signals derived from the abdominal wall[25]. The anterior abdominal wall is innervated by sensory neurons originated from the anterior rami of spinal nerves T6-L1 between the internal oblique and the transversus abdominis muscles[26].

Transversus abdominis plane (TAP) block is commonly applied for postoperative analgesia for abdominal surgeries. With the guidance of ultrasound or anatomical landmarks, local anesthetics can be injected into the space between the internal oblique and transversus abdominis muscles to block nerves T6-L1 [24, 27]. In this meta-analysis, TAP block from all the included studies was performed with the guidance of ultrasound, which could reduce the possible bias caused by the different guidance of TAP block.

Ropivacaine, bupivacaine and levobupivacaine are the commonly used local anesthetics in TAP block. Ropivacaine is widely used for postoperative pain due to a lower toxicity to cardiovascular and central nervous systems, a longer duration of block as well as a lower propensity for motor block at a low concentration[28]. De Oliveira et al. reported that the dose of local anesthetics for TAP block significantly affected the effects of postoperative analgesia[29]. Moeschler et al. found that the optimum volume of local anesthetics for unilateral TAP block was 15 ml[30]. A meta-analysis demonstrates that TAP block with 0.375% ropivacaine is able to reduce opioid consumption at 24 hours postoperatively. 0.375% ropivacaine is therefore recommended for TAP block[31]. The timing of TAP block is also varied. Preoperative period might be the optimal time for TAP block, because it could reduce early pain scores and opioid consumption comparing with postoperative period[29].

There are different approaches of TAP block in clinical practices, such as posterior, lateral, and subcostal TAP block. The posterior approach appears to be more effective than the lateral approach in patients undergoing lower abdominal surgeries[32]. For upper abdominal surgeries, the subcostal approach might be more beneficial than the posterior approach for postoperative analgesia[33]. Our results reveal that TAP block combined with dexamethasone can provide benefic effects on postoperative analgesia regardless of different approaches of TAP block. Subgroup analysis is theoretically need to be conducted to assess the effects of dexamethasone on different approaches of TAP block. However, it is not conducted in our current manuscript due to the insufficient sample size. For instance, the TAP block performed under subcostal approach was reported in only one study [16]. To date, no study has been conducted to compare the effects of dexamethasone between different approaches of TAP block. Thus, this may need to be determined in future trials.

Many studies demonstrate the analgesic efficacy and safety of corticosteroids for neuraxial and peripheral nerve block[34, 35]. Studies also indicate a promising effect of dexamethasone as an adjuvant in TAP block[19, 36, 37]. However, no significant prolongation of analgesia for TAP blocks was observed when dexamethasone added[7, 16], and no change for analgesic consumption[16]. Wegner et al. found only a slight and insignificant reduction in pain scores at 12 hours after surgery in the dexamethasone group. However, the TAP block was not performed by the same and experienced technical staff. Several patients probably were provided with “failed” blocks but still included in the study[7]. Unlike other included studies, the TAP block was performed by subcostal approach in Huang’s research[16]. Opioid consumption in this study was not available for current meta-analysis. The authors reported that sufentanil at dose of 5ug was given intravenously for two patients in the dexamethasone group and for one patient in the control group. Instead, parecoxib consumption was calculated, but no significant difference was found. The different administration approach was considered as a leading cause for the different outcomes.

In current meta-analysis, the doses of dexamethasone in included studies were varied, which might contribute to the heterogeneity. Theoretically, a meta-regression can be used to evaluate the optimum dose for analgesia. However, the meta-regression was not conducted due to the insufficient sample size and statistical power. El Sharnouby et al. suggested that the addition of 4 mg dexamethasone was equipotent to 8 mg dexamethasone for TAP block[19]. Unfortunately, the data in VAS scores was not available for the current meta-analysis and we failed to get the raw data from the authors.

VAS score is the most common method to evaluate pain severity and relief. Numeric differences of the VAS score can cause statistically significance, however, it may not be necessarily of clinical importance. Kelly et al. reported that the minimum clinically significant difference (MCSD) in VAS score was 0.9 (95%CI, 0.6 to 1.3). The MCSD in VAS score did not vary with gender, age, and cause of pain[38]. Our results in VAS score are basically in line with the above range, therefore, dexamethasone combined with TAP block is considered to be clinically significant.

Somatic pain and visceral pain were assessed separately in one included study[10]. Both somatic pain and visceral pain scores were lower in dexamethasone group. Because TAP block is applied directly to block somatic nerves, the analgesic effect on visceral pain may mainly result from dexamethasone. Systemic effects of dexamethasone may produce the analgesic actions[39, 40]. However, there are limited studies that investigate the analgesic effect of TAP block or dexamethasone, or the combination of them on visceral pain.

Similar to the TAP block, the analgesic effect of dexamethasone was suggested greater with preoperative administration than intraoperative[41]. Preoperative administration might be the optimal time to perform TAP block with dexamethasone. However, our subgroup analysis reveals revealed that there is no statistically significant difference in morphine consumption when TAP block with dexamethasone performed preoperatively. In contrary, when TAP block with dexamethasone was performed postoperatively, morphine consumption was reduced in the dexamethasone group. Hence, further studies are still needed to determine the optimal timing.

The precise mechanism for reinforced analgesic effect of corticosteroid is unknown. McCormack et al. suggests that corticosteroid can generate analgesia through their anti-inflammatory or immune-suppressive actions[42]. Other studies indicate that the analgesic effect of corticosteroid is due to their direct inhibition of ectopic neural discharge[43]. Many studies also indicate that dexamethasone induces perineural vasoconstriction which may reduce the absorption rate of local anesthetics[44, 45]. Pennington et al. suggests steroids may enhance the effects of local anesthetics through modulation of potassium channels [39].

Postoperative nausea and vomiting (PONV) is a common and distressing complication following surgery and anesthesia that may lead to dehydration, electrolyte imbalance, wound dehiscence and delayed hospital discharge. A variety of antiemetic drugs, such as droperidol, metoclopramide, ondansetron, dexamethasone, and cyclizine, have been used for the prevention and treatment of PONV[46, 47].

Dexamethasone is effective for PONV when administered intravenously at the dose of 4–12 mg[48, 49]. Several studies demonstrated that dexamethasone is more effective for PONV when given immediately before the induction of anesthesia than at the end of anesthesia[41, 50]. However, our results reveal that dexamethasone is effective for PONV when given both preoperatively and/or postoperatively.

The mechanism for the antiemetic effect of dexamethasone is not well clear. Corticosteroids exert their antiemetic action via prostaglandin antagonism is commonly accepted [51]. Others suggest that the antiemetic effect of dexamethasone may be related to the release of endorphins[52]. Sakae et al[52] found that perineural dexamethasone was more effective than intravenous when added to ropivacaine on the duration of ultrasound-guided interscalene brachial plexus blocks.

Two included studies[17, 19] concerned the complications of dexamethasone. Although a previous meta-analysis[41] reported that dexamethasone given intravenously significantly increased blood glucose levels during the first postoperative day, similar results were not found in the patients who received dexamethasone in these two studies. Different approaches of administration may account for these inconsistent results.

Initially, TAP block is performed based on anatomical landmarks. Complications include bowel hematoma, enlarged liver laceration, and transient femoral nerve palsy were reported [53]. Recently, with the guidance of ultrasound, complications of TAP block have been significantly reduced[54]. Previous studies suggest ultrasound-guided TAP block is a safe technique for postoperative pain management[55, 56]. In this meta-analysis, TAP block in included studies was all performed with ultrasound-guided technique, and no complication was reported.

Three limitations still exist in this meta-analysis. Firstly, only nine studies are included and thus the sample size is relatively small. Secondly, the conditions between included studies are varied, including type of surgery and anesthesia, the concentration and volume of local anesthetics, the dose of dexamethasone, the timing and approach of TAP block and the analgesia methods, which may increase the heterogeneity. Finally, different opioid probably affect the efficacy and duration of TAP block. Therefore, large samples, multicenter, randomized and controlled clinical trials are still needed to investigate the optimal strategy of TAP block combined with dexamethasone for postoperative analgesia.

## Conclusions

This meta-analysis demonstrates that dexamethasone added to local anesthetics in TAP block can decrease pain scores at 4, 6, 12h postoperatively (low or moderate quality evidence), increase the time to first request for additional analgesics postoperatively (moderate quality evidence), reduce morphine consumption (low quality evidence) and the incidence of PONV (high quality evidence). We recommend the routine use of a dexamethasone-local anesthetics TAP block as a part of multimodal analgesic regimen after abdominal surgeries to enhance the recovery process.

## Supporting information

S1 Appendix Search strategy used for PubMed. (DOCX)

S2 Appendix PRISMA checklist. (DOC)
