# Supplementary material for: Dexamethasone added to local anesthetics in ultrasound-guided transversus abdominis plain (TAP) block for analgesia after abdominal surgery: A systematic review and meta-analysis of randomized controlled trials
Source: PLoS One. 2019 Jan 8;14(1):e0209646. doi: 10.1371/journal.pone.0209646 (PMC6324803; doi:10.1371/journal.pone.0209646)
Supplement: S1 Appendix — (DOCX) [file pone.0209646.s001.docx]

**S1 Appendix. Search strategy used for PubMed**

| **Database** | **Group** | **Search Syntax** | **Records** |
| --- | --- | --- | --- |
| PubMed | 1 | (“Dexamethasone”[Mesh] OR “Dexamethasone”[Title/Abstract] OR “Hexadrol”[Title/Abstract]) OR “Oradexon”[Title/Abstract]) OR “Millicorten”[Title/Abstract]) OR “Maxidex”[Title/Abstract]) OR “Dexasone”[Title/Abstract]) OR “Hexadecadrol”[Title/Abstract]) OR “Methylfluorprednisolone”[Title/Abstract]) | **67166** |
|  | 2 | (“Transversus abdominis plane block”[Title/Abstract] OR “Transverse abdominis plane block”[Title/Abstract] OR “TAP block”[Title/Abstract]) | **678** |
|  | **#1 AND #2** | | **15** |
